# Oxidative Stress Regulates CDH3 Expression in Lung Cancer Cells via OGG1-Mediated SP1 Binding

**DOI:** 10.3390/antiox14030332

**Published:** 2025-03-11

**Authors:** Ying Ma, Jiarong Guo, Shichu Xu, Yanjun Hou, Feiyan Pan, Zhigang Guo

**Affiliations:** Jiangsu Key Laboratory for Molecular and Medical Biotechnology, College of Life Sciences, Nanjing Normal University, 1 Wen Yuan Road, Nanjing 210023, China; 191202035@njnu.edu.cn (Y.M.); guojr.stu@sibet.ac.cn (J.G.); 241202083@njnu.edu.cn (S.X.); houy@njnu.edu.cn (Y.H.)

**Keywords:** CDH3, OGG1, oxidative stress, gene regulation, transcription initiation

## Abstract

Oxidative stress, resulting from an imbalance between reactive oxygen species (ROS) production and antioxidant defenses, plays a crucial role in tumor development. Tumor cells often experience elevated oxidative stress due to rapid proliferation and unstable metabolism, leading to DNA damage. The enzyme 8-oxoguanine DNA glycosidase (OGG1) is central to repairing oxidative DNA damage, thereby maintaining genomic stability. In addition to its DNA repair function, OGG1 also plays a role in gene expression under oxidative stress. This study examined the expression pattern of cadherin-3 (CDH3), a cell adhesion protein associated with cancer metastasis and poor prognosis, under oxidative stress. Our findings showed that oxidative stress upregulated CDH3 expression, with OGG1 playing a pivotal role. Analysis of the *CDH3* promoter revealed SP1 binding sites, and ChIP-qPCR assays confirmed OGG1’s involvement in modulating SP1 binding. These results provided new insights into the regulation of CDH3 under oxidative stress and suggested potential therapeutic strategies targeting CDH3 in cancer treatment.

## 1. Introduction

Oxidative stress occurs when the production of oxidants exceeds the capacity of antioxidant systems, primarily due to elevated levels of reactive oxygen species (ROS) [1], which is one of the significant physiological responses to changes in cellular metabolism and the microenvironment. Tumor cells often exist in an environment of high oxidative stress due to their rapid proliferation and unstable metabolism [2]. Oxidative stress plays a complex, dual role in tumor initiation, progression, and metastasis [3]. Elevated ROS levels are not only crucial for cell signaling but can also induce oxidative damage to intracellular DNA, proteins, and lipids [4]. A notable example of oxidative DNA damage is the formation of 8-oxoguanine (8-oxoG), a highly mutagenic lesion that results from ROS-induced modifications to guanine [5]. The accumulation of 8-oxoG, particularly in GC-rich regions such as the SP1 transcription factor binding sites, has been shown to enhance gene transcription, thereby linking oxidative damage to alterations in gene expression [6]. To counteract the harmful effects of oxidative damage and preserve genomic stability and cell viability, tumor cells must activate efficient repair mechanisms. In this process, 8-oxoguanine DNA glycosidase (OGG1) plays a central role [7], which is responsible for recognizing and excising 8-oxoG [8]. Furthermore, recent studies have revealed that OGG1 interacts directly with the SP1 transcription factor. This interaction facilitates the SP1-mediated transcription of inflammation-related genes [9], suggesting a critical link between oxidative stress and the regulation of gene expression through OGG1. The dual role of OGG1 in DNA repair and transcriptional activation underscores its importance in the adaptive response of cells to oxidative stress [10,11,12,13]. However, additional downstream genes regulated by OGG1 under oxidative stress remain to be identified.

Cell adhesion molecules influence the migration, invasion, and metastasis of tumor cells by regulating the interactions between cells and the combination of cells and extracellular matrix [14]. Among these molecules, cadherin-3 (CDH3), also known as P-cadherin, is a key cell adhesion protein that belongs to a family of calcium ion-dependent cell adhesion proteins [15]. In normal epithelial tissues, CDH3 plays a crucial role in maintaining cell polarity and tissue integrity. However, in cancer, CDH3 expression is often dysregulated, leading to altered adhesion properties that promote tumor progression and metastasis [16]. CDH3 expression is significantly upregulated in various cancers, and is associated with poor prognosis and increased tumor invasiveness [17,18,19,20]. Due to its low expression in normal human tissues, CDH3 is considered an ideal therapeutic target for a range of malignant solid tumors [21]. Furthermore, CDH3 expression has been shown to be positively regulated by Kruppel-like transcription factor 4 (KLF4) in hepatocellular carcinoma [22]. Similarly, CCAAT/enhancer binding protein β (C/EBP β) is a transcription regulator of CDH3 in breast cancer [23,24]. However, the expression and regulatory mechanism of CDH3 under oxidative stress in lung cancer remain poorly understood.

Herein, we investigated the regulatory mechanism underlying CDH3 expression under oxidative stress and demonstrated that oxidative stress upregulates CDH3 expression, with OGG1 playing a key role in this process. Using JASPAR online software (https://jaspar.elixir.no/analysis, accessed on 26 October 2024), we analyzed the *CDH3* promoter region and identified multiple SP1 binding sites, with the region from −158 to −150 bp showing the highest binding affinity. ChIP-qPCR assays confirmed the binding of SP1 and OGG1 at this site under oxidative stress, highlighting the crucial role of OGG1 in facilitating the SP1-mediated regulation of CDH3 expression. Overall, this study elucidates the mechanism of CDH3 transcriptional regulation under oxidative stress and offers new insights for targeted CDH3-based therapeutic strategies.

## 2. Materials and Methods

### 2.1. Cell Culture and Treatment

The human non-small-cell lung cancer cell line A549 used in this study was obtained from American Tissue Culture Collection cell bank and cultured in RPMI 1640 Medium (KGL1501-500, KGI Biotechnology, Nanjing, China) containing 10% Fetal Bovine Serum (BC-SE-FBS08, SenBeiJia Biological Technology Co., Ltd., Nanjing, China) at 37 °C in a wet 5% CO_2_ incubator. Equal numbers of cells were seeded into six-well plates and cultured overnight. Following treatment with 30 μM H_2_O_2_ for 24 h, cells were collected for subsequent experiments.

### 2.2. Antibodies

The anti-SP1 (A19649), anti-E-cadherin (A19649), anti-Vimentin (A19649), anti-α-SMA (A19649), anti-GAPDH (A19056), HRP Goat Anti-Rabbit IgG (H + L) (AS014), and HRP Goat Anti-Mouse IgG (H + L) (AS003) antibodies were purchased from Abclonal (Wuhan, China). The anti-OGG1 (sc-376935) and anti-8-oxoG DNA Lesion (sc-130914) antibodies were purchased from Santa Cruz Biotechnology (Dallas, TX, USA). The antibodies against N-cadherin (22018-1-AP) and CDH3 (13773-1-AP) were purchased from Proteintech (Wuhan, China). Alexa Fluor^®®^ 594 Goat-Anti-Mouse (A-11005) was purchased from Life Technologies (Carlsbad, CA, USA).

### 2.3. Transcriptome Sequencing

After knocking down OGG1 in cells, samples were collected. The TRIzol reagent was then added to fully lyse cells. The RNA extraction, quality inspection, library construction, sequencing, and other processes were entrusted to Shanghai Meiji Biotechnology Co., Ltd. (Shanghai, China). Eukaryotic mRNA sequencing was performed on the Illumina NovaSeq 6000 sequencing platform (Illumina, San Diego, CA, USA), and library construction was carried out using the Illumina TruSeq™ RNA Sample Prep Kit method. HISAT2 (Version 2.1.0) was used for sequence alignment, StringTie (Version 2.1.2) for transcript assembly, and DESeq2 for differential expression analysis. Each sample generated approximately 51 million clean reads, and the mapped data for each sample contained approximately 48 million reads.

### 2.4. Cell Transfection

Cells were transfected with vectors using transfection reagent (T101-01, Vazyme, Nanjing, China). The shRNA sequence targeting CDH3 (Gene ID: 1001) was GCTCAAGTCTAATAAAGAT. The shRNA sequence targeting OGG1 (Gene ID: 4968) was GGAGTGGTGTACTAGCGGATC. A scrambled shRNA was used as the negative control, designated as shRNA—control. The overexpression of CDH3 was achieved by transfection with 3 μg CDH3 overexpression vector (NM 001793.6, Nanjing Corues Biotechnology Co., Ltd., Nanjing, China).

### 2.5. Overall Survival or Disease-Free Survival Analysis

GEPIA (Gene Expression Profiling Interactive Analysis; http://gepia.cancer-pku.cn/, accessed on 26 December 2024) was used for overall or disease-free survival analysis. The median gene expression value served as the group cutoff, separating samples with expression levels above (the high expression group) or below (the low expression group) the median.

### 2.6. Transwell Assay

Cells were seeded in the upper chamber of a transwell (Corning Inc., Corning, NY, USA) containing 200 μL serum-free medium, with 600 μL of culture medium containing 20% FBS added to the lower chamber. After 24 h, cells in the upper chamber were removed, and cells in the lower chamber were fixed with 4% paraformaldehyde for 15 min and stained with 0.1% crystal violet for 15 min. Images were captured from five randomly selected fields using an inverted microscope (Nikon, Tokyo, Japan).

### 2.7. Clone Formation Assay

Cells were seeded into 6-well plates. After 10–14 days, cells were fixed with 4% paraformaldehyde and stained with 0.1% crystal violet.

### 2.8. Growth Curve

Cells transfected with the sh-NC or sh-CDH3 vector were seeded into each well of a 96-well plate. Cell proliferation over one week was assessed using Cell Counting Kit-8 (K1018, APExBIO Technology LLC, Houston, TX, USA). Prior to measurement, 10 μL CCK-8 of solution was added to each well. The plates were then incubated for 1 h at 37 °C, and the absorbance at 450 nm was measured using a microplate reader.

### 2.9. Western Blotting

Cells were lysed in 1% SDS buffer with 1 mM PMSF and separated by SDS-PAGE. Proteins were transferred to PVDF membranes (03010040001, Roche, Basel, Switzerland) using the eBlot™ L1 system (L00686C, GenScript Biotech, Nanjing, China). After blocking with 5% skimmed milk for 2 h, membranes were incubated overnight at 4 °C with primary antibodies, followed by 2 h incubation with HRP-conjugated secondary antibodies. Detection was performed using ECL solution (FD8030, Hangzhou Fude Biotechnology Co., Ltd., Hangzhou, China) and imaging on a Tanon 4500 system (Tanon, Shanghai, China).

### 2.10. Real-Time Quantitative PCR (RT-qPCR) Analysis

Total cellular mRNA was extracted using the FastPure Cell/Tissue Total RNA Isolation Kit (RC112-01, Vazyme, Nanjing, China) according to the manufacturer’s instructions. Next, 1 μg of total RNA was reverse-transcribed into cDNA using HiScript II Q RT SuperMixR222-01, Vazyme, Nanjing, China. Each well in the qPCR assay contained 20 μL, including cDNA, AceQ qPCR SYBR Green Master Mix (high ROX master mix) (Q141-02, Vazyme, Nanjing, China), primers, and ddH_2_O. The CDH3 (Gene ID: 1001), OGG1 (Gene ID: 4968), and β-actin (Gene ID: 60) primers used in this study are listed in Table 1.

### 2.11. Immunofluorescence (IF) Assay

Cells were seeded in 12-well plates with slides and cultured overnight. After fixation with 4% paraformaldehyde for 10 min and permeabilization with 0.5% Triton X-100 for 15 min, cells were blocked with 3% BSA for 2 h. Following overnight incubation with primary antibodies at 4 °C, cells were treated with fluorescent secondary antibodies and stained with DAPI. Fluorescent images were captured using a Nikon confocal microscope (Nikon Instruments, Kawasaki, Japan).

### 2.12. Chromatin Immunoprecipitation (ChIP) Assay

The ChIP assay was performed using the ChIP Assay Kit (P2078, Beyotime, Shanghai, China) following the manufacturer’s instructions. Antibodies against SP1 and OGG1 were used to immunoprecipitate SP1 and OGG1 proteins, respectively. As a negative control, samples were treated with equal amounts of rabbit IgG (A7016, Beyotime, Shanghai, China) or mouse IgG (A7028, Beyotime, Shanghai, Shanghai). The DNA from immunoprecipitated samples and whole cell extracts was analyzed using real-time PCR (ChIP-qPCR). The primer sequences for the *CDH3* (Gene ID: 1001) promoter region detected by ChIP are listed in Table 2.

### 2.13. Statistical Analysis

All experiments were performed at least three times with at least three replicates in each experiment. The results were presented as the means ± SEM. Data from three independent experiments were analyzed statistically using Prism 8 software (GraphPad, La Jolla, CA, USA). *p* values were calculated using Student’s *t*-test. Differences with *p* < 0.05 were considered statistically significant.

## 3. Results

### 3.1. Identification of Downstream Molecules of OGG1 Through RNA Sequencing Analysis

**Figure 1:**
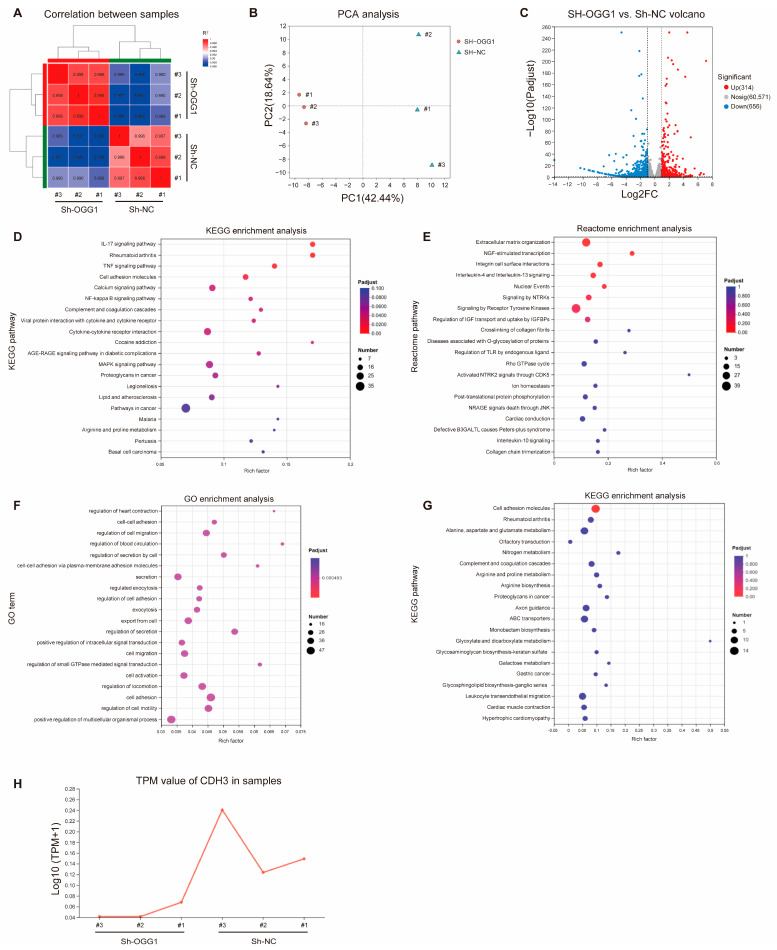
Transcriptome sequencing after the knockdown of OGG1 in A549 cells under oxidative stress. (**A**) The correlation analysis between samples. (**B**) The principal component analysis (PCA) between PC 1 and PC 2. (**C**) Volcanic map of differentially expressed genes. (**D**) KEGG enrichment analysis of DEGs. (**E**) Reactome enrichment analysis of DEGs. (**F**) GO enrichment analysis of significantly downregulated DEGs. (**G**) KEGG enrichment analysis of significantly downregulated DEGs. (**H**) Transcripts per million (TPM) value of CDH3 in samples.

Our previous research has demonstrated that OGG1 is crucial for the proliferation and metastasis of lung cancer cells under oxidative stress [13]. To identify additional downstream genes involved in the initiation and progression of lung cancer, RNA sequencing was performed on sh-NC and sh-OGG1 cells under oxidative stress. Correlation analysis confirmed good reproducibility within each group (Figure 1A,B). Following OGG1 knockdown, a total of 970 differentially expressed genes (DEGs) were identified, including 314 upregulated genes and 656 downregulated genes (Figure 1C). The Kyoto Encyclopedia of Genes and Genomes (KEGG) enrichment analysis and Reactome enrichment analysis of DEGs revealed significant enrichment in pathways related to cell adhesion molecules and extracellular matrix organization (Figure 1D,E). Furthermore, Gene Ontology (GO) and KEGG enrichment analyses of the significantly downregulated genes also indicated that these genes are primarily involved in cell adhesion and migration (Figure 1F,G), which is consistent with previous reports linking OGG1 to tumor cell migration. Notably, CDH3, a key adhesion molecule, was significantly downregulated (Figure 1H).

### 3.2. CDH3 Promotes Tumor Cell Proliferation and Migration

**Figure 2:**
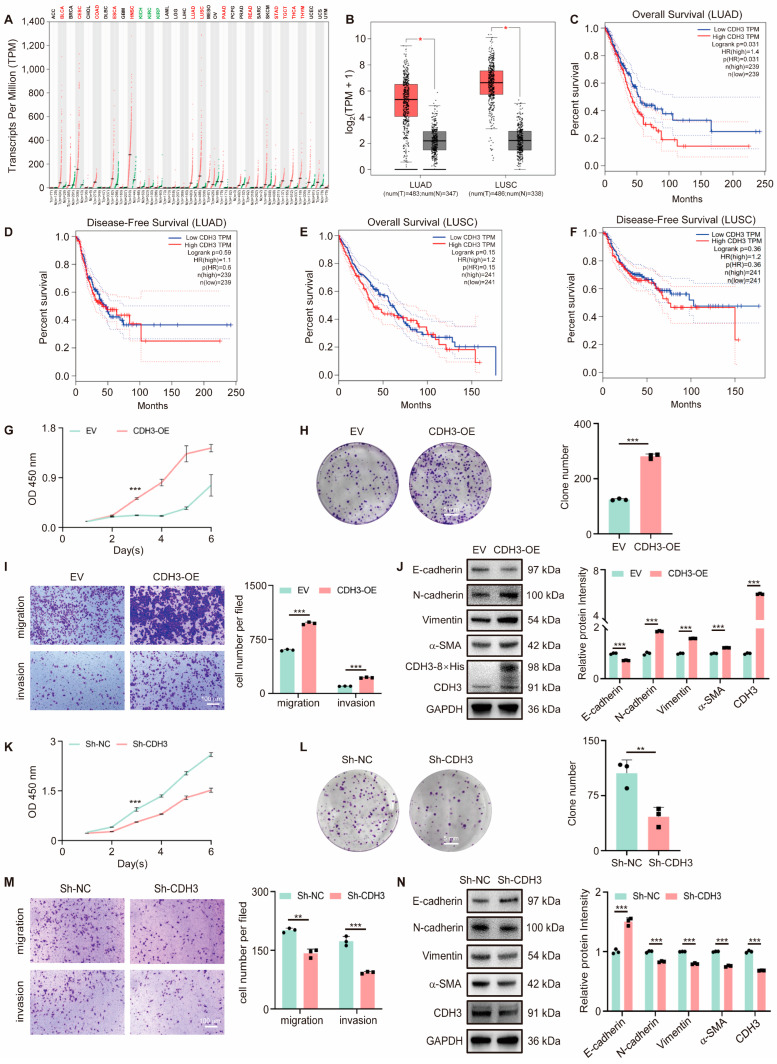
CDH3 is involved in tumor cell proliferation and migration. (**A**) Pan-cancer analysis of CDH3. Tumors with a significantly higher expression of CDH3 compared to paired normal tissues are labeled in red, while those with a significantly lower expression than paired normal tissues are labeled in green. (**B**) The CDH3 expression in LUAD and LUSC. The method for differential analysis was one-way ANOVA (* *p* < 0.05). (**C**,**D**) The overall survival (**C**) and disease-free survival (**D**) of patients with LUAD, stratified based on their CDH3 expression levels. Solid lines represent survival curves or disease-free survival curves respectively. Dashed lines represent 95% confidence intervals. (**E**,**F**) The overall survival (**E**) and disease-free survival (**F**) of patients with LUSC, stratified based on their CDH3 expression levels. Solid lines represent survival curves or disease-free survival curves respectively. Dashed lines represent 95% confidence intervals. (**G**) The growth curve of empty vector (EV) and CDH3-OE A549 cells. (**H**) The clone formation assay in EV and CDH3-OE A549 cells. Scale bar: 5 mm. Right, quantification of colony numbers. (**I**) Representative images of the transwell assay in EV and CDH3-OE A549 cells. Scale bar: 100 μm. Right, quantification of migrated cells. (**J**) Western blot analysis of epithelial-mesenchymal transition (EMT)-related proteins in A549 cells transfected with EV or CDH3-OE. Right, quantification of EMT markers. (**K**) The growth curve of A549 cells transfected with sh-NC or sh-CDH3. (**L**) The clone formation assay in A549 cells transfected with sh-NC or sh-CDH3. Scale bar: 5 mm. Right, quantification of colony numbers. (**M**) Representative images of the transwell assay in A549 cells transfected with sh-NC or sh-CDH3. Scale bar: 100 μm. Right, quantification of migrated cells. (**N**) Western blot analysis of EMT-related proteins in A549 cells transfected with sh-NC or sh-CDH3. Right, quantification of EMT markers. All experiments were performed with three biological replicates. The statistical significance was assessed using the unpaired Student *t*-test (** *p* < 0.01; *** *p* < 0.001).

Analysis of The Cancer Genome Atlas (TCGA) database reveals that CDH3 is widely and highly expressed in various cancers (Figure 2A), including lung cancer (Figure 2B). Furthermore, patients with a high CDH3 expression exhibited a shorter overall survival and disease-free survival compared to those with low CDH3 expression (Figure 2C–F). To further investigate CDH3’s role in lung cancer, overexpression and knockdown experiments were performed. CDH3 overexpression significantly enhanced cell proliferation and migration (Figure 2G–J), while its knockdown had the opposite effect (Figure 2K–N). Collectively, these findings suggest a strong association between CDH3 expression and tumor initiation and progression.

### 3.3. OGG1 Regulates CDH3 Expression

**Figure 3:**
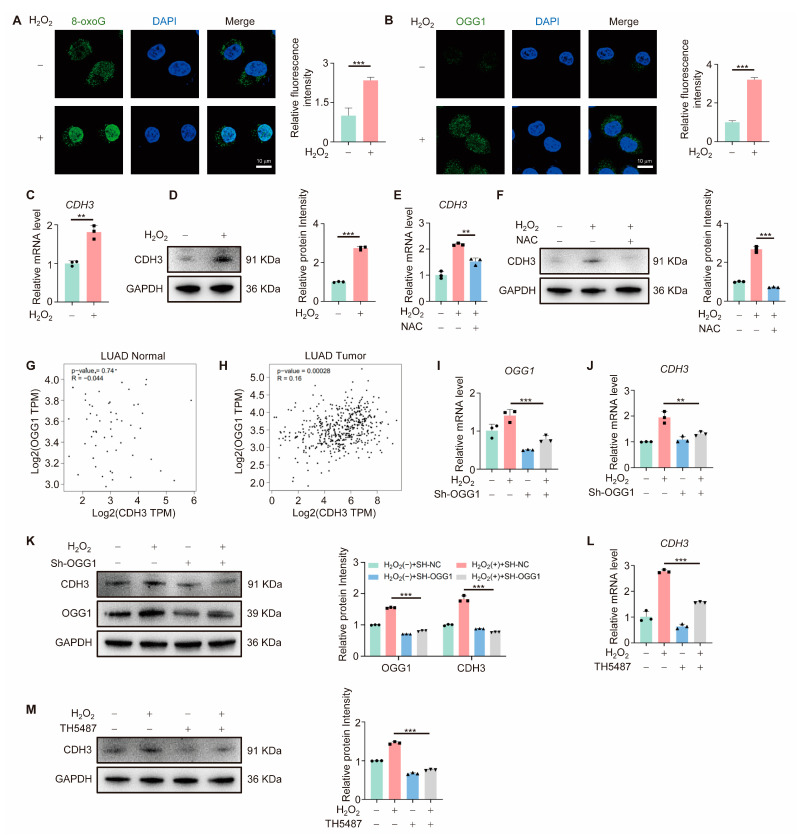
CDH3 is a new downstream target of OGG1. (**A**,**B**) Representative images of 8-oxoG (**A**) and OGG1 (**B**) in A549 cells treated with or without 30 μM H_2_O_2_ for 24 h, as detected by IF assay. Scale bar: 10 μm. Right, quantification of immunostaining for 8-oxoG or OGG1. (**C**,**D**) The mRNA (**C**) and protein (**D**) levels of CDH3 in A549 cells treated with or without 30 μM H_2_O_2_ for 24 h. Right, quantification of CDH3. (**E**,**F**) The mRNA (**E**) and protein (**F**) levels of CDH3 in A549 cells treated with 10 mM NAC for 24 h. Right, quantification of CDH3. (**G**,**H**) The correlation analysis between the expression of OGG1 and CDH3 in normal lung tissue (**G**) and LUAD tissue (**H**). (**I**–**K**) The mRNA (**I**,**J**) and protein (**K**) levels of OGG1 and CDH3 in A549 cells transfected with sh-NC or sh-OGG1. Right, quantification of OGG1 and CDH3. (**L**,**M**) The mRNA (**L**) and protein (**M**) levels of CDH3 in A549 cells treated with or without TH5487. Right, quantification of CDH3. All experiments were performed with three biological replicates. The statistical significance was assessed using the unpaired Student *t*-test (** *p* < 0.01; *** *p* < 0.001).

OGG1, a key enzyme in DNA oxidative damage repair, regulates transcription through binding to 8-oxoG [12,25]. To investigate its role in CDH3 regulation under oxidative stress, cells were treated with H_2_O_2_. As shown in Figure 3A,B, H_2_O_2_ treatment elevated intracellular 8-oxoG levels and induced OGG1 nuclear import, confirming oxidative stress induction. CDH3 expression, both at the mRNA and protein levels, was significantly elevated under oxidative stress (Figure 3C,D), and NAC (N-acetylcysteine—a potent antioxidant) treatment reduced CDH3 expression, confirming its responsiveness to ROS signaling (Figure 3E,F). Next, GEPIA database analysis revealed no correlation between OGG1 and CDH3 expression in normal lung tissue (Figure 3G), but a significant positive correlation in LUAD tissue (Figure 3H). To further explore the role of OGG1 in regulating CDH3 under oxidative stress, A549 cells were transfected with an sh-OGG1 plasmid to knock down OGG1. The results showed that OGG1 knockdown significantly reduced CDH3 expression under oxidative stress (Figure 3I–K), as did treatment with the OGG1 inhibitor TH5487 (Figure 3L,M), which was consistent with transcriptome sequencing data. Taken together, these findings indicate that CDH3 is a potential downstream target of OGG1.

### 3.4. CDH3 Is Downstream of SP1

**Figure 4:**
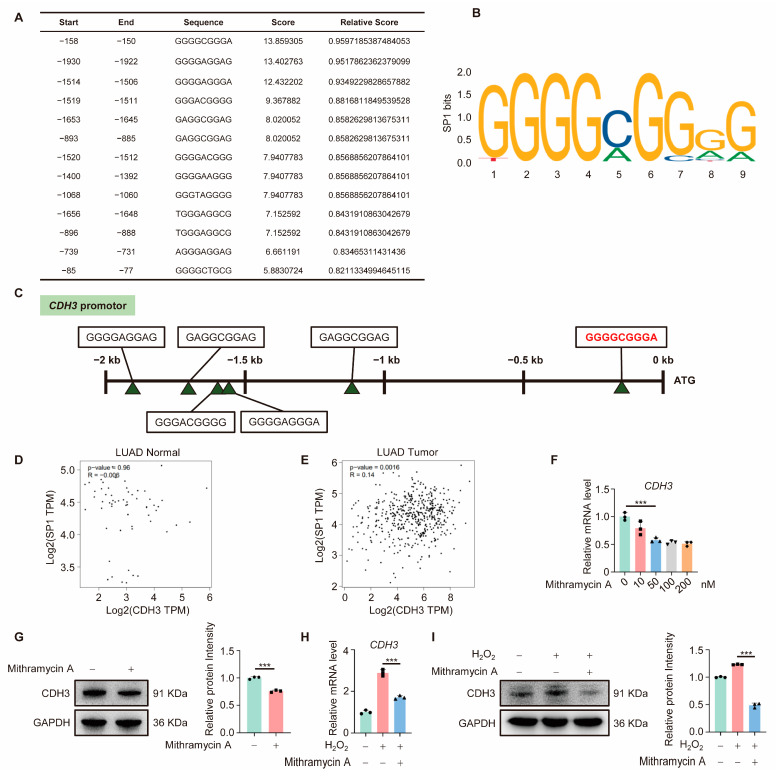
SP1 regulates CDH3 expression. (**A**) Analysis of the *CDH3* promoter. SP1 binding sites in the *CDH3* promoter region were predicted using the JASPAR database. (**B**) SP1 sequence logo. Sequence logos were created online using JASPAR online tools. (**C**) Position distribution of SP1 binding sites in the *CDH3* promoter region with a “Score” greater than 8. The sequence with the highest binding potential is marked in red. (**D**,**E**) The correlation analysis between the expression of OGG1 and CDH3 in normal lung tissue (**D**) and LUAD tissue (**E**). (**F**) The mRNA level of CDH3 in A549 cells treated with different concentrations of Mithramycin A. (**G**) The protein level of CDH3 in A549 cells treated with or without 50 nM Mithramycin A. Right, quantification of CDH3. (**H**,**I**) The mRNA (**H**) and protein (**I**) levels of CDH3 in A549 cells treated with or without 50 nM Mithramycin A. Right, quantification of CDH3. All experiments were performed with three biological replicates. The statistical significance was assessed using the unpaired Student *t*-test (*** *p* < 0.001).

To investigate the transcription factor involved in OGG1-mediated CDH3 expression, the first 2 kb of the *CDH3* promoter region was analyzed using the JASPAR online tool (https://jaspar.genereg.net/, accessed on 26 October 2024). This analysis identified multiple potential SP1 binding sites (Figure 4A) and the sequence logo of SP1 used in this study was shown in Figure 4B. The region from −158 to −150 bp exhibited the highest binding potential (Figure 4A,C). Previous studies have demonstrated that SP1 directly interacts with OGG1 [9], suggesting that SP1 may serve as a key transcription factor in the regulation of CDH3 expression by OGG1. Furthermore, GEPIA database analysis showed no correlation between SP1 and CDH3 expression in normal lung tissue (Figure 4D), but a significant positive correlation in LUAD tissue (Figure 4E). To further explore SP1’s role in regulating CDH3, we treated cells with Mithramycin A, a known SP1 inhibitor. As the concentration of Mithramycin A increased, CDH3 mRNA levels decreased (Figure 4F) and the protein level of CDH3 was also reduced (Figure 4G). Moreover, Mithramycin A treatment reduced CDH3 expression under oxidative stress, indicating that SP1 is a critical transcription factor for CDH3 expression (Figure 4H,I). Collectively, these findings suggest that CDH3 is downstream of SP1 under oxidative stress.

### 3.5. OGG1 Is Involved in SP1 Binding in the CDH3 Promoter Region

**Figure 5:**
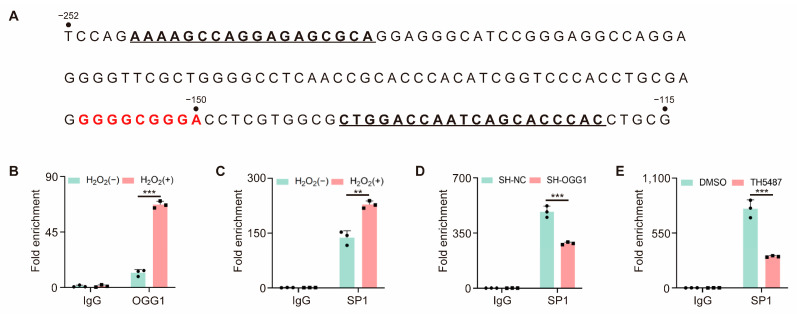
OGG1 is involved in the binding of SP1 to the *CDH3* promoter under oxidative stress. (**A**) *CDH3* promoter’s partial sequence. The potential SP1 binding sites from −158 to −150 bp are marked in red. The underline indicates the primers used for ChIP-qPCR. (**B**,**C**) ChIP-qPCR analysis of OGG1 (**B**) and SP1 (**C**) enrichment at the *CDH3* promoter under oxidative stress. (**D**) ChIP-qPCR analysis of SP1 enrichment at the *CDH3* promoter under oxidative stress in OGG1 knockdown cells. (**E**) ChIP-qPCR analysis of SP1 enrichment at the *CDH3* promoter under oxidative stress in TH5487-treated cells. All experiments were performed with three biological replicates. The statistical significance was assessed using the unpaired Student *t*-test (** *p* < 0.01; *** *p* < 0.001).

To investigate the role of OGG1 in the regulation of CDH3 by SP1 under oxidative stress, we performed ChIP-qPCR assays to assess the enrichment of OGG1 and SP1 in the *CDH3* promoter region (Figure 5A). As shown in Figure 5B,C, the binding of both OGG1 and SP1 to the *CDH3* promoter increased under oxidative stress. However, following OGG1 knockdown and the inhibition of OGG1 binding activity, SP1 enrichment was significantly reduced under oxidative stress (Figure 5D,E). These results suggest that OGG1 contributes to the SP1-mediated regulation of CDH3 expression under oxidative stress.

## 4. Discussion

Tumor cells are frequently exposed to elevated levels of ROS due to their heightened metabolic and proliferative activities [26]. During this process, OGG1 plays a crucial role in maintaining genome stability and cell viability in tumor cells by efficiently repairing oxidative DNA damage [27]. Recent studies have uncovered the involvement of OGG1 in the regulation of gene expression. OGG1 facilitates tumor metastasis by assisting the transcription factor NF-κB in regulating the expression of SYT7 [13]. This regulatory function enables OGG1 to contribute to the adaptation of tumor cells to oxidative stress, thereby influencing cell survival and proliferation. A further exploration of OGG1’s regulatory mechanisms and its role across different tumor types could provide new insights and potential strategies for cancer prevention and treatment. Therefore, identifying more downstream targets of OGG1 is essential. Notably, RNA-Seq analysis following OGG1 knockdown revealed a significant reduction in tumor cell adhesion-related pathways, which suggested that OGG1 is closely linked to tumor initiation and progression. By unraveling this complex relationship, we can gain a deeper understanding of OGG1’s role in tumor development and potentially identify novel therapeutic targets.

Cell adhesion molecules play pivotal and multifaceted roles in tumor metastasis [28]. These molecules are critical in regulating the migration and invasion of tumor cells by modulating cell–cell adhesion, influencing the EMT, regulating cell–matrix interactions, and mediating various signaling pathways [29,30]. Consistent with these studies, the overexpression or knockdown of CDH3 leads to corresponding changes in EMT markers. However, morphological changes in the cells were not significantly observed. In this study, CDH3 overexpression or knockdown was achieved through plasmid transfection. Cellular morphological changes are generally a relatively long-term process, often requiring prolonged observation and more stable gene expression. Therefore, although CDH3 plays an important role in the EMT process, the short-term increase or decrease in expression induced by plasmid transfection has a limited effect on inducing significant morphological changes. In future studies, lentiviral infection will be used to establish stable CDH3 overexpression or knockdown cell lines to further investigate the long-term effects of CDH3 on cell morphology. Next, we observed that oxidative stress upregulated the expression of CDH3, while knockdown OGG1 significantly reduced this upregulation, suggesting that OGG1 is involved in the regulation of CDH3 expression. Furthermore, analysis of the *CDH3* promoter region identified several potential SP1 binding sites, with the region from −158 to −150 bp exhibiting the highest binding potential. ChIP analysis confirmed the enrichment of SP1 and OGG1 in this region under oxidative stress. Notably, when OGG1 was knocked down or its binding activity was inhibited, the binding of SP1 to this region was markedly reduced, highlighting the essential role of OGG1 in promoting SP1 enrichment in the *CDH3* promoter region. However, due to inter-individual differences driven by somatic mutations and genomic rearrangements, which may result in significant variability, the use of a single cell line (A549) in this study presents certain limitations. Another cell line could potentially yield different results. The same mechanisms in other cancer cells require further investigation.

Oxidative stress-induced DNA damage, particularly the formation of 8-oxoG at GC-rich SP1 binding sites, has been shown to promote the activation of downstream gene transcription [6], which is consistent with our experimental findings. The OGG1-driven transcription initiation model proposed by Ba et al. provides important insights into the impact of oxidative damage on transcriptional processes [9]. Under oxidative stress, the binding of OGG1 to 8-oxoG facilitates the interaction between transcription factors and promoters, representing a part of the cellular adaptive response to oxidative stress. Similarly, our study reveals that OGG1 enhances the accessibility of the SP1 binding site within the *CDH3* promoter region. Furthermore, treatment with TH5487, which disrupts the interaction between OGG1 and 8-oxoG, significantly reduced SP1 enrichment at the *CDH3* promoter and subsequently downregulated CDH3 expression, indicating that OGG1’s role in transcriptional regulation is closely linked to its DNA repair activity. However, under steady-state conditions, normal cells typically maintain low levels of ROS, resulting in a lack of sustained accumulation of 8-oxoG in the promoter regions of target genes; as such, the regulatory effect of OGG1 on CDH3 expression may be limited. The precise role of OGG1 in regulating CDH3 expression in normal cells warrants further investigation.

In conclusion, this study elucidates the mechanism by which OGG1 is involved in the binding of SP1 at the *CDH3* promoter region, thereby regulating its expression under oxidative stress. Cell adhesion molecules were significantly downregulated following OGG1 knockdown, which contributed to understanding OGG1’s role in tumor initiation and progression and offering a basis for potential OGG1-targeted therapies. Furthermore, the study demonstrated that both OGG1 and SP1 are enriched in the −158 to −150 bp region of the *CDH3* promoter under oxidative stress, providing new insights into the regulation of CDH3 expression in tumors. This also paves the way for therapeutic strategies targeting CDH3. Notably, OGG1 deletion led to reduced SP1 binding at this site, underscoring OGG1’s role as an upstream regulator of gene expression under oxidative stress and its significance in tumor development. Overall, the relationship between DNA oxidative damage signals and tumorigenesis is multifaceted. This study offers a novel perspective on oxidative stress-mediated tumor progression and identifies potential therapeutic targets.

## Figures and Tables

**Table 1:** The primer sequences for RT-qPCR.

Name	Sequence (5′-3′)
qPCR-CDH3-F	ATCATCGTGACCGACCAGAAT
qPCR-CDH3-R	GACTCCCTCTAAGACACTCCC
qPCR-OGG1-F	CACACTGGAGTGGTGTACTAGC
qPCR-OGG1-R	CCAGGGTAACATCTAGCTGGAA
qPCR-β-actin-F	CATGTACGTTGCTATCCAGGC
qPCR-β-actin-R	CTCCTTAATGTCACGCACGAT

**Table 2:** The primer sequences for ChIP-qPCR.

Name	Sequence (5′-3′)
ChIP-CDH3-F	AAAAGCCAGGAGAGCGCA
ChIP-CDH3-R	GTGGGTGCTGATTGGTCCAG

## Data Availability

The data presented in this study are available on request from the corresponding author.
